# *Notes from the Field:* Prevalence of Previous Dengue Virus Infection Among Children and Adolescents Aged 7–16 Years — American Samoa, September–October 2023

**DOI:** 10.15585/mmwr.mm7331a3

**Published:** 2024-08-08

**Authors:** Sandra Kiplagat, Noelle Tavale, Adam Konrote, Astrid M. Johansson, Angelynn Papu, Janice Perez-Padilla, Forrest K. Jones, Hans Desale, Annette F. Ilimaleota, Jacki M. Tulafono, Mark Delorey, Emma Jones, Emi Chutaro, Janet Camacho, Freddy Medina, Rafael Tosado-Acevedo, Jorge L. Munoz-Jordan, Gabriela Paz-Bailey, Laura E. Adams, Motusa Tuileama Nua, Joshua M. Wong, Scott Anesi

**Affiliations:** ^1^Epidemic Intelligence Service, CDC; ^2^Division of Vector-Borne Diseases, National Center for Emerging and Zoonotic Infectious Diseases, CDC; ^3^American Samoa Department of Health; ^4^Laboratory Leadership Service, CDC; ^5^Pacific Island Health Officers Association, Honolulu, Hawaii.

**Summary**

**What is already known about this topic?**

In 2021, CDC’s Advisory Committee on Immunization Practices recommended dengue vaccination for children and adolescents aged 9–16 years with laboratory-confirmed previous dengue virus (DENV) infection who live in areas with frequent or continuous dengue transmission. To reduce false positive prevaccination screening results, dengue vaccination should be implemented only if ≥20% of age-eligible persons have previously been infected with DENV.

**What is added by the report?**

During 2023, a school-based dengue serosurvey in American Samoa found evidence of previous infection in 60% of persons aged 9–16 years.

**What are the implications for public health practice?**

DENV seroprevalence in American Samoa exceeds the minimum 20% threshold established for the introduction of recommended dengue vaccines to reduce the risk for hospitalization and severe dengue in seronegative children and adolescents. Dengue vaccines could be part of a broader strategy to reduce illness and death.

Dengue is a vectorborne disease caused by four dengue viruses (DENVs) and is transmitted through the bite of infected *Aedes* species mosquitoes. Dengue transmission in American Samoa is classified as frequent or continuous,* with 660 confirmed cases reported to CDC’s national arboviral surveillance system during the 2016–2018 outbreak (*1*). Infection usually confers lifelong immunity to the infecting virus serotype but only offers temporary protection against other DENVs. Because a second infection with DENV is more likely to result in severe illness^†^ than is a first or postsecondary infection, in 2021, the Advisory Committee on Immunization Practices recommended the Dengvaxia dengue vaccine (Sanofi Pasteur, Inc.) for persons aged 9–16 years with laboratory confirmation of previous DENV infection living in areas with frequent or continuous dengue transmission (*2*). Dengvaxia clinical trials demonstrated protection for persons with previous DENV infection but identified increased risk for severe dengue and hospitalization among persons without previous infection who were vaccinated (*2*). 

Because most DENV infections are asymptomatic, subclinical, and rarely laboratory-confirmed (*3*), identification of previous DENV infection to determine vaccine eligibility requires a positive result from a serologic test meeting CDC-recommended performance standards.^§^ However, tests are more likely to result in incorrect positive (false positive) results and more likely to misclassify seronegative persons in low seroprevalence geographic areas than in high seroprevalence areas. To reduce the chances of mistakenly vaccinating children and adolescents with false positive test results, vaccine introduction is advised only in areas where ≥20% of the eligible children and adolescents have previously had dengue. The 20% seroprevalence corresponds to a positive predictive value of ≥90% for a test with minimum sensitivity of 75% and minimum specificity of 98% (*2*,*4*). Dengue seroprevalence among children and adolescents in American Samoa is unknown. To determine whether the minimum 20% threshold for vaccination implementation had been reached, a serosurvey was conducted in American Samoa during September–October 2023. This activity was reviewed by CDC, deemed not research, and conducted in accordance with applicable federal law and CDC policy.^¶^

## Investigation and Outcomes

### Study Design and Analysis

To guide decisions on dengue vaccine implementation, on August 8, 2023, the American Samoa Department of Health requested assistance from CDC to determine the prevalence of previous DENV infection among school-age children and adolescents through a school-based serosurvey. Seven of 36 public schools were randomly selected through a single-stage cluster sampling design stratified by school type.** All students in grades 3–10 enrolled in the selected schools were invited to participate. Students with a signed parental permission form were tested using the CTK Biotech OnSite dengue immunoglobulin G rapid test^††^ (sensitivity = 89.6%; specificity = 95.7%) (*5*). Seroprevalence estimates were computed using survey design weights and adjusting for test sensitivity and specificity.

### Seroprevalence Findings

During September–October 2023, a total of 2,267 students were invited to participate in the serosurvey, and 887 (39%) received testing. The median participant age was 11 years (range = 7–16 years). More than one half (54%) of participants were female. Among tested students, 492 (56%) received positive results for dengue immunoglobulin G and 371 (42%) received negative results; results for 24 (3%) students were uninterpretable. The estimated seroprevalences among females and males were 61% and 56%, respectively. The estimated seroprevalence among all students aged 7–16 years was 59% (95% CI = 47%–71%) and was 60% (95% CI = 48%–72%) among those age-eligible for vaccination (i.e., those aged 9–16 years). Dengue seroprevalence was lowest among children aged 8 years (46%; 95% CI = 32%–60%) (Table).

**TABLE:** Estimated dengue virus immunoglobulin G seroprevalence among children and adolescents aged 7–16 years — American Samoa, September–October 2023

Characteristic	No. of participants tested*	No. of participants with positive test results	Estimated IgG seroprevalence, % (95% CI)^†^
**Total, aged 7–16 yrs**	**887**	**492**	**59 (47–71)**
**Age-eligible for vaccination, 9–16 yrs**	**767**	437	60 (48–72)
**Age, yrs**			
7	**17**	9	84 (26–100)
8	**103**	46	46 (32–60)
9	**100**	52	59 (37–80)
10	**124**	71	53 (25–81)
11	**115**	72	67 (50–84)
12	**127**	66	53 (34–71)
13	**135**	85	72 (56–88)
14	**102**	56	58 (28–88)
15	**61**	32	55 (17–94)
16	**3**	3	100 (—)
**Sex^§^**			
Female	**475**	273	61 (49–74)
Male	**411**	219	56 (44–74)

**Abbreviation**: IgG = immunoglobulin G.

* Twenty-four participants received uninterpretable test results: males (14); and children and adolescents aged 7 years (three), 8 years (two), 9 years (two), 10 years (two), 11 years (six), 12 years (four), 13 years (three), 14 years (one), and 15 years (one).

^†^ Seroprevalence was estimated using survey weights and adjusted for sensitivity and specificity.

^§^ One participant did not specify their sex.

## Preliminary Conclusions and Actions

Dengue seroprevalence is approximately 60% among persons age-eligible (9–16 years) for dengue vaccination in American Samoa, exceeding the minimum threshold of 20% established for the introduction of recommended dengue vaccines to reduce the risk for severe dengue and hospitalization while minimizing the risk associated with vaccine administration to persons who have not been previously infected. Seroprevalence is high among all age groups, indicating widespread previous exposure to DENV and potential risk for future outbreaks as well as associated secondary cases among persons previously infected. In American Samoa, dengue vaccines could be part of a broader strategy for dengue control that would also include mosquito control at home, mosquito bite prevention measures, training of health care providers to recognize and treat dengue, and improving laboratory capacity to strengthen surveillance to effectively reduce illness and death.

## Footnotes

* Frequent or continuous dengue risk is defined as evidence of more than 10 dengue cases in ≥3 of the previous 10 years. https://www.cdc.gov/dengue/areas-with-risk/index.html

† Severe dengue is defined as dengue with any of the following clinical manifestations: severe plasma leakage leading to shock or fluid accumulation with respiratory distress; severe bleeding; or severe organ impairment such as hepatitis (elevated transaminases ≥1,000 IU/L), impaired consciousness, or heart impairment. https://www.cdc.gov/dengue/hcp/clinical-signs/index.html

§ CDC recommends prevaccination screening tests to have ≥75% sensitivity, ≥98% specificity, ≥90% positive predictive value, and ≥75% negative predictive value. In areas with frequent or continuous transmission, qualifying laboratory tests for vaccination include positive reverse transcription–polymerase chain reaction or nonstructural protein 1 test results. https://www.cdc.gov/dengue/hcp/vaccine/testing.html

¶ 45 C.F.R. part 46.102(l)(2), 21 C.F.R. part 56; 42 U.S.C. Sect. 241(d); 5 U.S.C. Sect. 552a; 44 U.S.C. Sect. 3501 et seq.

** The schools comprised five elementary schools (kindergarten–8th grade) and two high schools (9th–12th grade).

†† The CTK Biotech test was conducted for public health purposes and not for determining individual vaccine eligibility. Testing for individual vaccine eligibility would require the two-test algorithm (https://www.cdc.gov/dengue/hcp/vaccine/testing.html) and require that it be performed in a laboratory meeting requirements to conduct and report results for clinical purpose, which was not available in American Samoa at the time the survey was conducted.

## References

[1] Ryff KR, Rivera A, Rodriguez DM, Epidemiologic trends of dengue in U.S. territories, 2010–2020. MMWR Surveill Summ 2023;72(No. SS-4):1–12. 10.15585/mmwr.ss7204a1 37192141 PMC10208330

[2] Paz-Bailey G, Adams L, Wong JM, Dengue vaccine: recommendations of the Advisory Committee on Immunization Practices, United States, 2021. MMWR Recomm Rep 2021;70(No. RR-6):1–16. 10.15585/mmwr.rr7006a1 34978547 PMC8694708

[3] Shankar MB, Rodríguez-Acosta RL, Sharp TM, Tomashek KM, Margolis HS, Meltzer MI. Estimating dengue under-reporting in Puerto Rico using a multiplier model. PLoS Negl Trop Dis 2018;12:e0006650. 10.1371/journal.pntd.0006650 30080848 PMC6095627

[4] Mac VV, Wong JM, Volkman HR, Notes from the field: prevalence of previous dengue virus infection among children and adolescents—U.S. Virgin Islands, 2022. MMWR Morb Mortal Wkly Rep 2023;72:288–9. 10.15585/mmwr.mm7211a4 36927833 PMC10027406

[5] Medina FA, Vila F, Adams LE, Comparison of the sensitivity and specificity of commercial anti-dengue virus IgG tests to identify persons eligible for dengue vaccination. medRxiv [Preprint posted online April 21, 2024]. 10.1101/2024.04.19.24306097 PMC11481482 39194193

